# Analysis of Operational Effects of Bus Lanes with Intermittent Priority with Spatio-Temporal Clear Distance and CAV Platoon Coordinated Lane Changing in Intelligent Transportation Environment

**DOI:** 10.3390/s25082538

**Published:** 2025-04-17

**Authors:** Pei Jiang, Xinlu Ma, Yibo Li

**Affiliations:** 1 School of Traffic and Transportation, Chongqing Jiaotong University, Chongqing 400074, China; jp@mails.cqjtu.edu.cn (P.J.); liyb@mails.cqjtu.edu.cn (Y.L.); 2 Chongqing Key Laboratory of Intelligent Integrated and Multidimensional Transportation System, Chongqing Jiaotong University, Chongqing 400074, China

**Keywords:** intelligent transportation, connected and automated vehicles, coordinated lane changing, traffic simulations, intelligent control

## Abstract

Bus lanes with intermittent priority (BLIP) are designed to optimize road resource allocation. The advent of connected and automated vehicles (CAVs) promotes the implementation of BLIP. However, it is crucial to find an effective method to intermittently grant right-of-way to CAVs. In this paper, we introduce a BLIP method with spatio-temporal clear distance (BLIP-ST) and a CAV control method in an intelligent transportation environment. When CAVs access BLIP-ST, the constraints of the moving gap between buses are considered. When CAVs leave BLIP-ST, coordination with the nearest CAV platoon on the adjacent lane is considered to cope with situations where CAVs cannot find the appropriate space. Then, the proposed method was simulated by an open boundary cellular automaton model. The results showed that with the same inflow, a CAV-sharing bus lane could significantly improve road traffic efficiency, and it is the most significant when the CAV penetration rate is medium, with the average road speed increasing from 6.67 km/h to 30.53 km/h. Meanwhile, when the CAV penetration rate is medium, BLIP-ST operates with the best efficiency at different strategies. This was due to the fact that when the penetration rate is too high, BLIP-ST is excessively occupied, which affects public transportation priority. When the penetration rate is too low, BLIP-ST cannot be fully utilized. In addition, regardless of the penetration rate of CAV, CAV platoon collaborative lane changing is better than single CAV collaborative lane changing in terms of improving road traffic efficiency and can increase the average road speed by 8–19%.

## 1. Introduction

**Figure 1:**
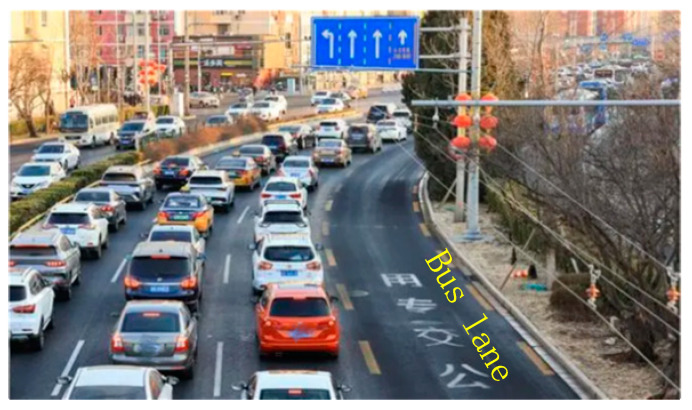
Current operation of bus lanes.

Bus lanes can improve the efficiency of bus travel, but lower lane utilization may result in a waste of road resources, as shown in Figure 1. Segmented shared bus lanes are an innovative way to optimize roadway use and enhance bus service reliability [1]. The intermittent bus lane (IBL), proposed in 1996 by Viegas, notifies other vehicles not to enter the bus lane when a bus has arrived at the entrance of the road through a Horizontal Road Lighting System (HILS) or Variable Message Signs (VMS) [2]. IBL was implemented in Lisbon, Portugal; Melbourne, Australia; and Lyon, France, with various studies supporting its effectiveness. On the basis of IBL, Eichler et al. proposed a Bus Lane with Intermittent Priority (BLIP) and allowed private vehicles and buses to share bus lanes, with VMS controlling the entry and exit of private vehicles at appropriate times [3]. Carey et al. conducted simulation tests on BLIP by VISSIM in Oregon, USA [4]. Similarly. Xie et al. demonstrated that dynamic lane allocation strategies significantly enhance bus transit performance in BLIP [5]. Leclercq evaluated the impact of intermittent priority (BLIP) strategies—which dynamically allocate bus lanes to general traffic during idle periods—on capacity and travel times in signalized corridors [6].

Early studies on BLIP laid the groundwork, while later scholars have substantially advanced the theoretical and practical understanding of this strategy.

Wu et al. proposed BLIP with a fixed clear distance and then identified optimal clear distances through VISSIM simulations [7,8]. Wu analyzed the relationship between the compliance of private vehicles and traffic efficiency in BLIP with clearance distance using the cellular automaton model (CA model) [9]. Ma et al. divided the bus lane into multiple static clear areas of equal length and improved the spatial utilization of the dedicated lane by combining different numbers of clear areas while ensuring bus priority [10]. Chiabaut et al. evaluated the effects of an intermittent bus lane strategy on traffic conditions after a detailed presentation of the 350 m case [11]. Some scholars have also studied the application of the concept of BLIP to other lanes, e.g., Chen [12].

Existing research has primarily focused on human-driven vehicles (HVs) in BLIP. While BLIP shows considerable promise, its widespread implementation encounters significant challenges in managing HV behavior. However, recent advancements in vehicle-road communication technologies and the emergence of connected and automated vehicles (CAVs) may overcome these limitations, leveraging CAV’s inherent advantages in controllability, safety, reliability, and operational efficiency. Nevertheless, research on BLIP applications in mixed-traffic environments with intelligent and connected vehicles remains scarce.

Wu et al. established a CA model of BLIP within a vehicular network environment, analyzing factors such as the constant clear distance, traffic flow density, and bus departure intervals applicable to BLIP [13]. Chen et al. developed a theoretical model to prove that allocating CAVs to dedicated bus lanes could increase the efficiency of rapid transit, employing SUMO (Simulation of Urban Mobility) to simulate and compare different allocation strategies [14]. Pang et al. introduced a CAV lane-borrowing control strategy that satisfied both safety requirements and lane-changing incentives [15]. Dong et al. proposed a dynamic CAV bus-lane borrowing method utilizing the CA model to optimize lane-changing timing [16].

Furthermore, numerous studies have investigated mixed traffic flow. For example, FATEMEH et al. modeled CAVs and HVs while considering driving behavior [17]. TALEBPOUR et al. developed a game theory lane-changing model for CAVs [18], and SALA et al. used probabilistic theory to estimate the CAV distribution in mixed flows, finding that CAV platoon strategies enhance road capacity [19]. Zhou et al. established a CA (cellular automata) modeling framework to simulate heterogeneous traffic flow and proposed a strategy to improve road capacity by combining the interaction of heterogeneous elements [20]. Qin analyzed the mixed flow stability from a macroscopic perspective considering different vehicle following characteristics [21], and Liu et al. proposed a mixed traffic flow model architecture in order to investigate the influence of an adaptive cruise control CACC system on the operating characteristics of mixed traffic flow on a multi-lane highway [22]. It can be seen that there is relatively little research on mixed traffic flow models that consider buses. Liang et al. established a heterogeneous traffic flow model that considered bus and CAV platoon characteristics [23]. Shan et al. proposed a trajectory optimization method that allows some CAVs to enter the bus lane and optimize their own trajectories based on the known bus trajectories [24]. Yuan proposed a two-stage nonlinear control model to optimize bus trajectories in mixed traffic [25]. Li et al. utilized signal timing to optimize bus lane usage under a partially connected vehicle environment [26]. In addition, some scholars focused on the CAV platoon. Liu et al. proposed an adaptive protocol for synchronized merging in the cyclic communication scenario [27].

In conclusion, for most of the strategies for reusing bus lanes, although the strategies or methods vary, the core concept is that vehicles a certain distance in front of the bus need to leave the bus lane. Although this strategy theoretically ensures the absolute priority of buses, when the traffic flow in the ordinary lane is significant, the vehicles that need to leave the bus lane cannot find the appropriate space to change lanes in time to return to the ordinary lane, thus blocking the operation of buses. ZHAO et al. pointed out that bus lanes with time-division multiplexing strategies could achieve optimal traffic allocation under low bus flow environments, while bus priority was difficult to effectively guarantee with high saturation and dense bus arrivals [28]. Although the issue has been raised that the vehicles that need to leave the bus lane cannot find the appropriate space to change lanes in time, it has not yet been resolved. Therefore, this study proposes a control method that considers CAV platoon cooperative lane changing to cope with situations where CAV cannot find the appropriate space, combined with the background of mixed traffic flow in the smart grid environment.

The structure of this paper is as follows:

(1) The basic assumptions and notation are presented in Section 2.
(2) In Section 3, we introduce a BLIP method based on the spatio-temporal clear distance (BLIP-ST) in Section 3.1 and establish the integrated heterogeneous traffic flow cellular automata model in Section 3.2. Then, we propose a CAVs borrowing bus lane control method considering the moving gap constraint in Section 3.3 and a CAV platoon collaborative lane-changing method in Section 3.4. When a target CAV that is within the clear distance of the bus cannot find the appropriate space to change lanes in time, the nearest CAV or CAV platoon on the adjacent lane will provide space by changing speeds.
(3) The proposed models and method are validated through numerical simulations in Section 4.
(4) We discuss the simulation results and compare the road operation performance before and after implementation across different strategies in Section 5.
(5) We provide a conclusion in Section 6.

## 2. Assumptions and Notations

### 2.1. Assumptions

Before proceeding to develop the model, the following assumptions underpin our scenarios and settings for the heterogeneous traffic flow and bus lane reuse problem:

(1) The traffic system consists of three vehicle types: HVs, CAVs, and human-driven buses, with each category exhibiting consistent performance parameters.
(2) The scenario is confined to a road segment, ensuring that lane-changing behavior does not impact arrival at the destination. CAV borrowing and leaving behavior adhere to the instructions of the control center with a 100% obedience rate. Considering that the controlled CAV is between two buses and has a small range, the communication delay is not considered.
(3) Leveraging V2X infrastructure, all connected and automated vehicles (CAVs) and the central control system maintain real-time awareness of surrounding traffic conditions.
(4) Communication delays or losses are not explicitly considered.

Based on these assumptions, this paper is organized as follows: Section 2 describes the setup and diagram of the BLIP-ST problem. Section 3 models heterogeneous traffic flow and lane changing based on an improved CA model. Section 4 presents numerical simulations. Section 5 reports the results of the numerical experiments. Finally, Section 6 summarizes the findings of the paper.

### 2.2. Notations

Before methodology, the notations involved in the text are interpreted, including vehicle information, road segment information, model parameters, and so on. Notations used throughout the paper are summarized in Table 1.

## 3. CAV Control Strategy and Modeling

### 3.1. BLIP-ST

**Figure 2:**
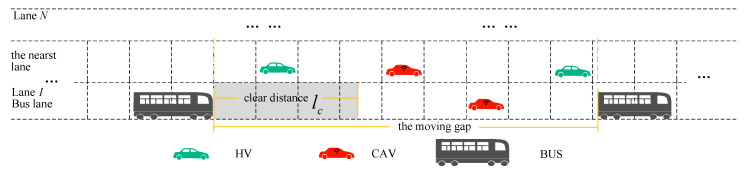
BLIP-ST diagram.

In this section, we define the clear distance to claim more reuse space, as shown in Equation (1). The clear distance of each bus varies according to Equation (1), with different buses traveling at different speeds with different clear distances. When a CAV is within the bus’s clear distance, it is required to leave the bus lane. Otherwise, the CAV may continue to travel in the bus lane. Clear distance $l_{c}(t)$ is shown in Figure 2.

(1) $$\begin{array}{ll} l_{c}(t)= & d_{bus,safe}(t)+L_{\mathrm{CAV}}\\ & =\tau_{\mathrm{BUS}}v_{bus}(t)-\frac{v_{bus}{\left(t\right)}^{2}}{2b_{\mathrm{BUS}}}{+L}_{\mathrm{CAV}} \end{array}$$

Here, $v_{bus}(t)$ is the speed of the bus, and other variable explanations can be found in Table 1.

### 3.2. Modeling

We conducted numerical simulations under an integrated heterogeneous traffic flow cellular automata model, which facilitates the dynamic display of the spatial and temporal evolution of traffic flows and clearly grasps the movement state of each vehicle [23]. The rules of vehicle movement are shown in Equations (2)–(5). The acceleration and deceleration rule are shown in Equations (2) and (3). The randomization rule is shown in Equation (4), and the movement rule is shown in Equation (5).

(2) $$\begin{array}{ll} v_{i}(t+\Delta t)= & (\alpha_{i}+\beta_{i})\min\left(v_{i}(t)+a_{i}\Delta t,v_{\max},\frac{d_{i}(t)-d_{i,safe}(t)}{\Delta t}\right)\\ & +\gamma_{i}\min\left(v_{i}(t)+a_{\mathrm{CAV}}\Delta t,v_{\max},\frac{d_{i}(t)-d_{i,safe}(t)}{\Delta t}+v_{i+1}(t)\right)\\ \mathrm{if}\;d_{i}(t)>v_{i}(t)\Delta t \end{array}$$

Here, $d_{i,safe}(t)=\tau v_{i}(t)-\frac{v_{i}{\left(t\right)}^{2}}{2b_{i}}$; here, $b_{i}=\left\{b_{\mathrm{BUS}},b_{\mathrm{HV}}\right\}$, $\tau =\left\{\tau_{\mathrm{BUS}},\tau_{\mathrm{HV}},0\right\}$. When the vehicle is a CAV, τ takes the value 0. $a_{i}\in \left\{a_{\mathrm{BUS}},a_{\mathrm{HV}}\right\}$.

(3) $$\begin{array}{l} v_{i}(t+\Delta t)=\left(\alpha_{i}+\beta_{i}\right)\max\left\{k_{1}v_{i}(t),v_{i}(t)+b_{i}\right\}+\gamma_{i}\max\left\{k_{1}v_{i+1}(t+\Delta t),v_{i}(t)+b_{\mathrm{CAV}}\Delta t\right\}\\ \mathrm{if}\;d_{i}(t)\leq v_{i}(t)\;\Delta t \end{array}$$

$$\mathrm{if}\;d_{i}(t)>d_{i,safe}(t),\;k_{1}=1,\;\mathrm{else}\;k_{1}=-1$$

(4) $$\begin{array}{l} v_{i}(t+\Delta t)=\max((v_{i}(t)-1),0)\\ \mathrm{if}\;p>p_{\mathrm{rand}} \end{array}$$

(5) $$x_{i}(t+1)=x_{i}(t)+v_{i}(t+1)\Delta t$$

In this study, we focused on the processes of CAVs’ collaborative lane changing when the target CAV could not find the appropriate space to leave the bus lane in time. Thus, regardless of the number of road lanes, our research focuses on the bus lane and its adjacent lanes.

### 3.3. CAVs’ Borrowing Bus Lane Control Method

**Figure 3:**
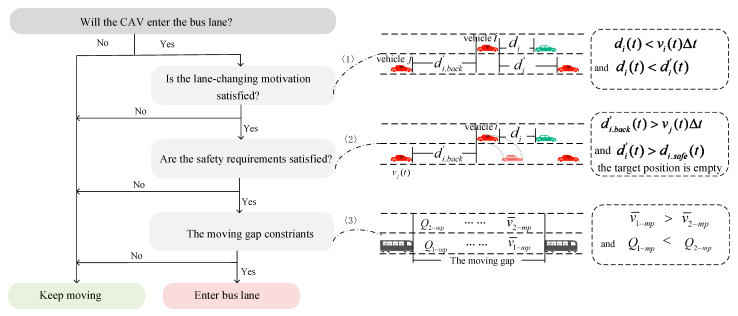
Flowchart of CAV borrowing BLIP-ST.

Following the BLIP-ST rule, the control method of CAVs’ borrowing bus lane is shown in Figure 3.

Lane-changing motivations are first evaluated when the target CAV i is not able to travel in the current lane at the current speed. The longitudinal gap between the target CAV i and the nearest leader is less than the gap to the nearest leader in the destination lane (the bus lane). This constraint ensures that the lane change is meaningful, as illustrated in Figure 3<1>.

Next, the CAV i could not affect the normal operation of the nearest follower in the destination lane and could not collide with the nearest leader in the destination lane. Additionally, the target position after lane changing must be unoccupied, as shown in Figure 3<2>.

Subsequently, we defined the moving gap that was formed by buses, as shown in Figure 2. The moving gap constraints contain the average speed and number of vehicles in the gap. In Figure 3<3>, $Q_{1-mp}$ is the number of vehicles in the bus lane in the moving gap. $Q_{2-mp}$ is the number of vehicles in the adjacent lane in the moving gap. ${\overset{-}{v}}_{1-mp}$ is the average speed in the bus lane in the moving gap. ${\overset{-}{v}}_{2-mp}$ is the average speed in the nearest lane in the moving gap.

### 3.4. CAVs Leaving Bus Lane Control Method

**Figure 4:**
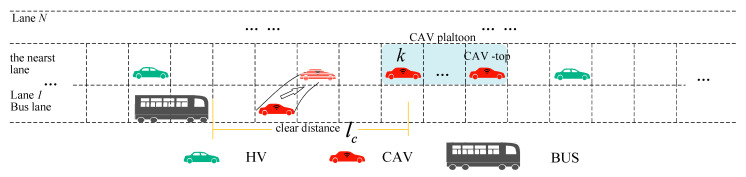
Schematic diagram of CAV leaving BLIP-ST.

**Figure 5:**
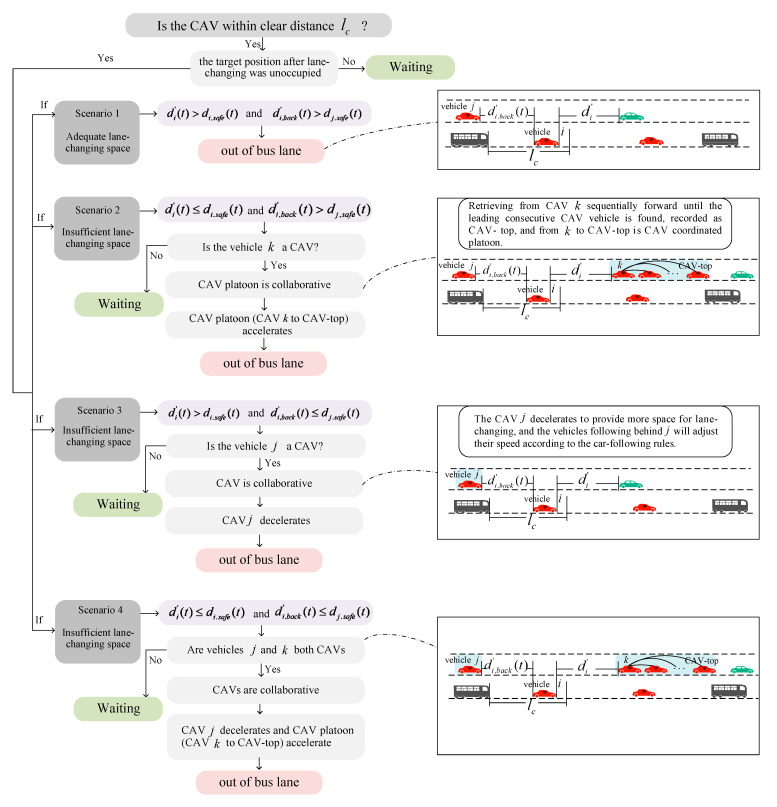
Flowchart of CAV leaving BLIP-ST diagram.

When CAV i enters the $l_{c}$ of the bus, it is required to leave the bus lane, as shown in Figure 4. The flowchart of CAVs leaving the bus lane under BLIP-ST rules is shown in Figure 5. There are four scenarios for leaving the bus lane.

Firstly, when CAV i was requested to leave the bus lane, the target position after lane-changing should be unoccupied.

In scenario 1, the target CAV i can leave the bus lane directly, and the target CAV i would not collide with the nearest follower and the nearest leader in the destination lane.

In scenario 2, the target CAV i cannot leave the bus lane directly. The target CAV i would not collide with the nearest follower in the destination lane but would collide with the nearest leader in the destination lane (an insufficient lane-changing space from the nearest leader). Thus, the nearest leader CAV or CAV platoon in the destination lane should provide space by changing speeds. Next, we analyzed the process of CAV platoon collaborative lane changing.

When vehicle k was HV, due to the uncontrollability of HVs, the target CAV i had to wait for the next opportunity. When vehicle k was a CAV (as shown in Figure 4), we retrieved from k sequentially until the leading consecutive CAV was found, recorded as CAV-top. k to CAV-top comprises the CAV collaborative lane-changing platoon. According to the assumptions, CAVs were randomly distributed, so the number of the CAV platoon was different at different times for different target CAVs. That is, CAV-top is variable, and CAV-top≥k. Then, the CAV platoon accelerates to provide the lane-changing space for the target CAV i. The collaborative lane-changing formula in scenario 2 is shown in Equation (6)

(6) $$\begin{array}{l} v_{\mathrm{CAV}-\mathrm{top}}(t+\Delta t)=\alpha_{\mathrm{CAV}-\mathrm{top}}\min\left(v_{\mathrm{CAV}-\mathrm{top}}(t)+a_{\mathrm{CAV}}\Delta t,v_{\max}^{\mathrm{CAV}},\frac{d_{\mathrm{CAV}-\mathrm{top}}(t)-d_{\mathrm{CAV}-\mathrm{top},safe}(t)}{\Delta t}\right)\\ \cdots \cdots \\ v_{k+1}(t+\Delta t)=\gamma_{k+1}\min\left(v_{k+1}(t)+a_{\mathrm{CAV}}\Delta t,v_{\max}^{\mathrm{CAV}},\frac{d_{k+1}(t)-d_{k+1,safe}(t)}{\Delta t}+v_{k+2}(t)\right)\\ v_{k}(t+\Delta t)=\gamma_{k}\min\left(v_{k}(t)+a_{\mathrm{CAV}}\Delta t,v_{\max}^{\mathrm{CAV}},\frac{d_{k}(t)-d_{k,safe}(t)}{\Delta t}+v_{k+1}(t)\right) \end{array}$$

Here, CAV-top≥k.

In scenario 3, the target CAV i would not collide with the nearest leader in the destination lane but would collide with the nearest follower in the destination lane (an insufficient lane-changing safe distance from the nearest follower). That is, CAV i could not change lanes directly, and the nearest follower CAV in the destination lane would providing space by changing speed. When vehicle j was an HV, the target CAV i had to wait for the next opportunity. When vehicle j was a CAV, CAV j should decelerate to provide the lane-changing space for the target CAV i. The formula in scenario 3 is shown in Equation (7).

(7) $$v_{j}(t+\Delta t)=\max\left(v_{j}(t)+b_{\mathrm{CAV}}\Delta t,\;0\right)$$

In scenario 4, the space from the nearest follower and the nearest leader in the destination lane were both not insufficient. When CAV i is required to leave the bus lane in scenario 4, collaborative strategies in scenario 2 and scenario 3 should be used simultaneously.

## 4. Numerical Simulations

**Figure 6:**
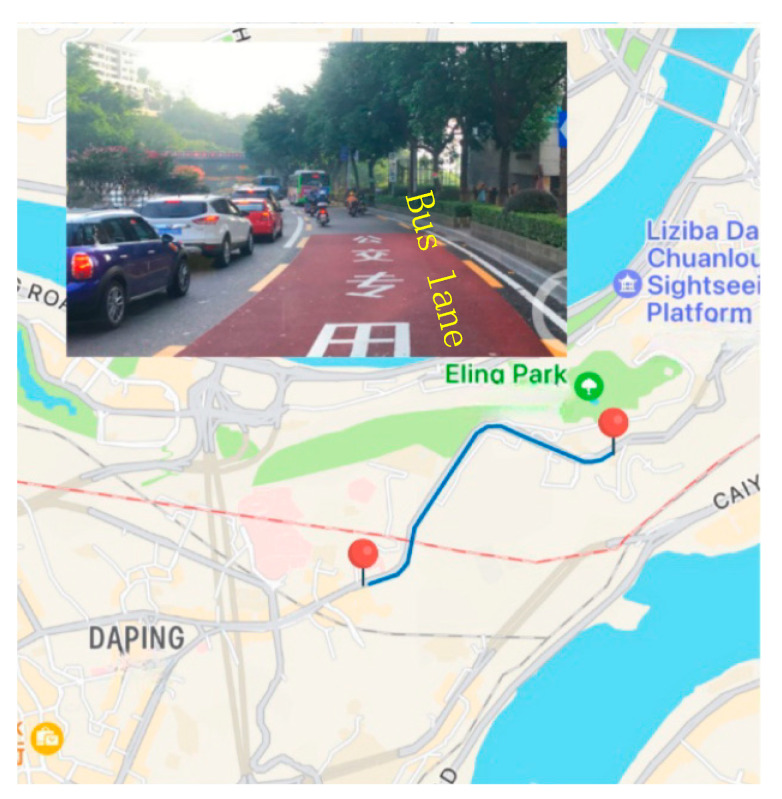
The selected road section in the direction of “Da ping—E ling”.

In this section, according to the above movement rules, we adopt open boundary conditions, which can simulate the situation where vehicles freely enter and exit, thus more accurately reflecting the actual traffic on the roads. Vehicles enter the road from the left boundary and leave via the right boundary. From a realistic view, the left boundary determines the inflow, and the right boundary determines the capacity [29]. Cars enter the road with a probability $p_{\mathrm{in}}$ at $v_{\max}$ and leave the road with a probability $p_{\mathrm{out}}$. The mathematical relationship between inflow and $p_{\mathrm{in}}$ can be represented by Equation (6) [30]. We selected a 2 km road section in the direction of “Da ping—E ling” during the morning peak hour of the Yangtze River 1st Road, Yuzhong District, Chongqing City, as depicted in Figure 6, and the experimental information is shown in Table 2. We measured the average inflow in the general lanes at the start of the road section, which was 1800 veh/h.

(8) $$Q_{\mathrm{in}}(p_{\mathrm{in}})=\frac{p_{\mathrm{in}}-{p_{\mathrm{in}}}^{v_{\max}+1}}{1-{p_{\mathrm{in}}}^{v_{\max}+1}}$$

Here, $Q_{\mathrm{in}}(p_{\mathrm{in}})$ is the inflow with $p_{\mathrm{in}}$.

**Table 2:** Relevant information regarding the data collection section.

Location	Nature	Time	Average Flow
Yangtze River 1st Road, Yuzhong District, Chongqing	Urban two-lane section	8:00–9:00	1800 veh/h

Then, we calculated $p_{\mathrm{in}}=0.5$ using Equation (8). In the bus lane, 18 bus lines passed through the investigation point. The bus frequency, determined using survey data from a downtown morning peak scenario, was four vehicles per minute. Simulation parameters are detailed in Table 3. Our research focused on the bus lane and the processes of CAVs collaborative lane-changing in the adjacent lane, so we simulated the bus lane and its adjacent lane.

## 5. Results and Discussion

In this section, we analyze the operational effects of three strategies, BLIP-ST(BS), BLIP-ST with a moving gap (BS-MP), and BLIP-ST with CAV platoon collaborative lane changing (BS-CPC) in different flows. A detailed explanation of the four strategies is given in Table 4. According to the open boundary conditions, cellular automata model, and $p_{\mathrm{in}}=0.5$, we control the flow by adjusting $p_{\mathrm{out}}$.

### 5.1. Free Flow Situation

**Figure 7:**
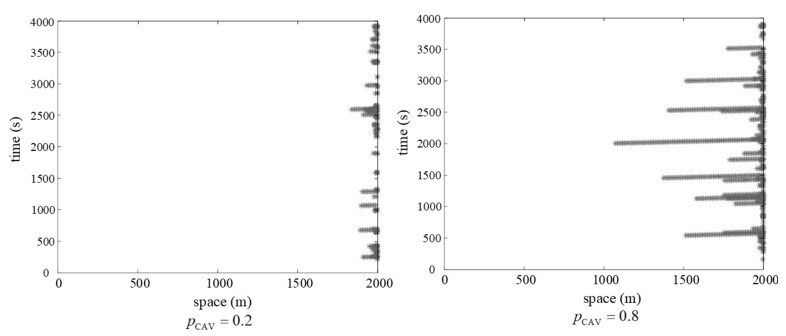
The time–space diagram of CAVs in bus lane in $p_{\mathrm{out}}=0.4$.

When the system is in free flow, it is unnecessary for CAVs to use the bus lane, which is verified through simulation. We set $p_{\mathrm{out}}=0.4$ and $p_{\mathrm{CAV}}=0.2$, $p_{\mathrm{CAV}}=0.8$, respectively. The duration of the simulation was 4000 s. The time–space diagram of CAVs in the bus lane is shown in Figure 7, which shows that when the road is free ($p_{\mathrm{in}}=0.5$ and $p_{\mathrm{out}}=0.4$), regardless of whether $p_{\mathrm{CAV}}$ is high or low, CAVs rarely enter the bus lane. Therefore, bus lane sharing cannot be effectively utilized when the road is in free flow.

### 5.2. Congestion Situation

In order to effectively utilize the bus lane, we set $p_{\mathrm{in}}=0.5$ and $p_{\mathrm{out}}=0.2$ by comparing the three strategies and the situation without any strategy (none of the strategies, NS).

**Figure 8:**
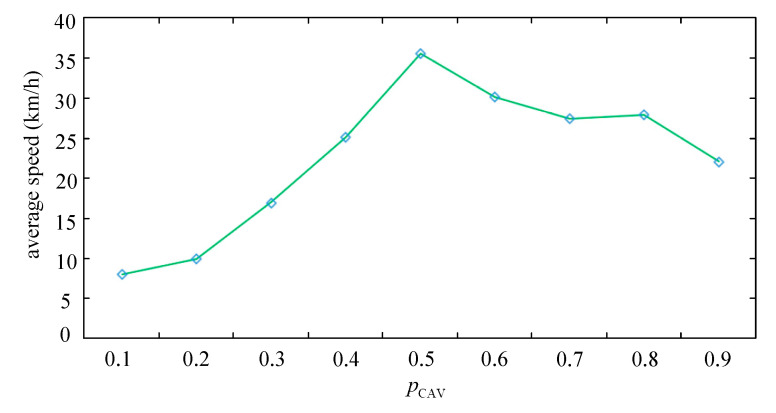
The average speeds of at different $p_{\mathrm{CAV}}$.

The first 3000 time steps are discarded to reduce the negative effect of the transient time. The results are obtained from 3001 to 4000 time steps. The average speeds under CAV platoon collaborative lane changing are shown Figure 8 at different $p_{\mathrm{CAV}}$. The performances of average speed indicated that road access is best at $p_{\mathrm{CAV}}=0.5$.

$p_{\mathrm{CAV}}=0.2$, $p_{\mathrm{CAV}}=0.5$, and $p_{\mathrm{CAV}}=0.8$ were chosen to represent a low CAV penetration rate, medium CAV penetration rate, and high CAV penetration rate, respectively. We discuss the simulations under the different strategies at different $p_{\mathrm{CAV}}$.

**Figure 9:**
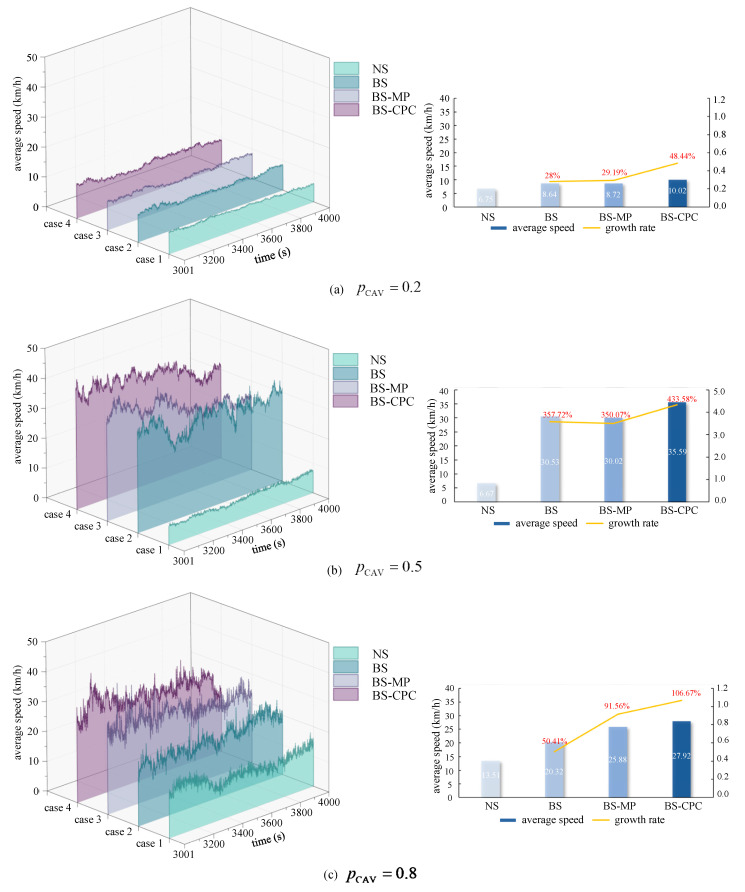
Road average speed under 4 strategies with different $p_{\mathrm{CAV}}$.

Figure 9 illustrates the average speeds under the different strategies. The results indicate that the average speeds in BS, BS-MP, and BS-CPC are significantly higher than in NS under different $p_{\mathrm{CAV}}$. Additionally, the performance of BS-CPC consistently achieved the highest average speed compared to other strategies.

When $p_{\mathrm{CAV}}=0.2$ (seen in Figure 9a), fewer CAVs reuse the bus lane. Specifically, CAVs reused the bus lane only 64 times within the selected 1000 timesteps at $p_{\mathrm{CAV}}=0.2$. At $p_{\mathrm{CAV}}=0.2$, the average speeds in BS, BS-MP, and BS-CPC were 8.64, 8.72, and 10.02, respectively. The average speed of NS is about 28–48.44% less than BS, BS-MP, and BS-CPC. On the other hand, BS-CPC had a 15% higher average speed than BS-MP, which suggests that at a low CAV penetration rate, CAV platoon cooperative lane changing is more effective than single CAV cooperation.

When $p_{\mathrm{CAV}}=0.5$ (seen in Figure 9b), the number of CAVs and HVs on the road is almost the same. The increase in the average speed in NS (6.67 km/h) is not significant compared to $p_{\mathrm{CAV}}=0.2$. But the average speed in BS, BS-MP, and BS-CPC increased the most, all exceeding 30 km/h. We noticed that the average speeds in BS and BS-MP are almost the same. By analyzing the data, we found that when the average speed of the moving gap on the bus lane was close to the average speed of the bus lane, the moving gap constraint was invalidated. That is because we applied the ceiling function to the velocity in simulations, and according to the rules of BS and BS-MP, the lane-changing constraints under BS and BS-MP were almost the same in this particular case. On the other hand, the average speed of BS-CPC is about 19% higher than that of BS-MP, which proves that the CAV platoon cooperative lane-changing effect is optimal under a medium CAV penetration rate.

When $p_{\mathrm{CAV}}=0.8$ (seen in Figure 9c), the average speed on the road exceeded 20 km/h in BS, BS-MP, and BS-CPC. Compared to $p_{\mathrm{CAV}}=0.5$, the performance was worse. Because more CAVs entered the bus lane, when congestion occurred, CAVs could not leave the bus lane promptly, leading to increased congestion and a reduction in the overall speed of the road section. Under NS, the average speed raised to 13.51 km/h at $p_{\mathrm{CAV}}=0.8$, more than twice the average speed at $p_{\mathrm{CAV}}=0.2$ or $p_{\mathrm{CAV}}=0.5$. This indicated that a high penetration rate of CAVs significantly improves vehicle speed in NS. On the other hand, the average speed in BS-CPC is still 8% higher than that in BS-MP, which also indicates that even in scenarios with high CAV penetration rates, CAV platoon coordinated lane-changing is still more effective than a single CAV.

**Figure 10:**
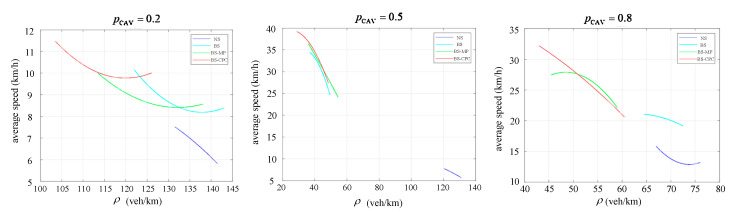
Diagram of the relationship between average speed and density.

**Figure 11:**
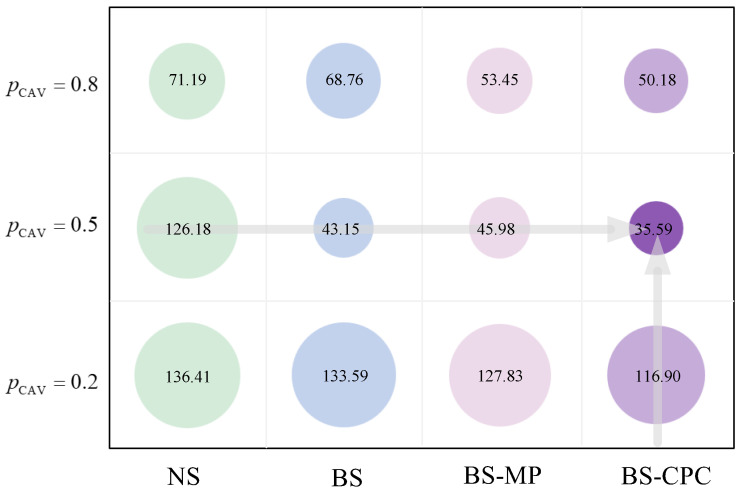
Average density bubble chart.

Next, we analyzed the relationship between the average speed and density under $p_{\mathrm{CAV}}=0.2$, $p_{\mathrm{CAV}}=0.5$, and $p_{\mathrm{CAV}}=0.8$, with the fit curves shown in Figure 10. ρ indicates density. It can be seen that the access performance was best at $p_{\mathrm{CAV}}=0.5$ under a constant flow, with the lowest density and highest average speed. When $p_{\mathrm{CAV}}=0.8$, it also improved the access effect of the road compared to $p_{\mathrm{CAV}}=0.2$ but was poorer than $p_{\mathrm{CAV}}=0.5$. The same conclusion can be drawn from Figure 11, which indicates the average density for $p_{\mathrm{CAV}}$.

**Figure 12:**
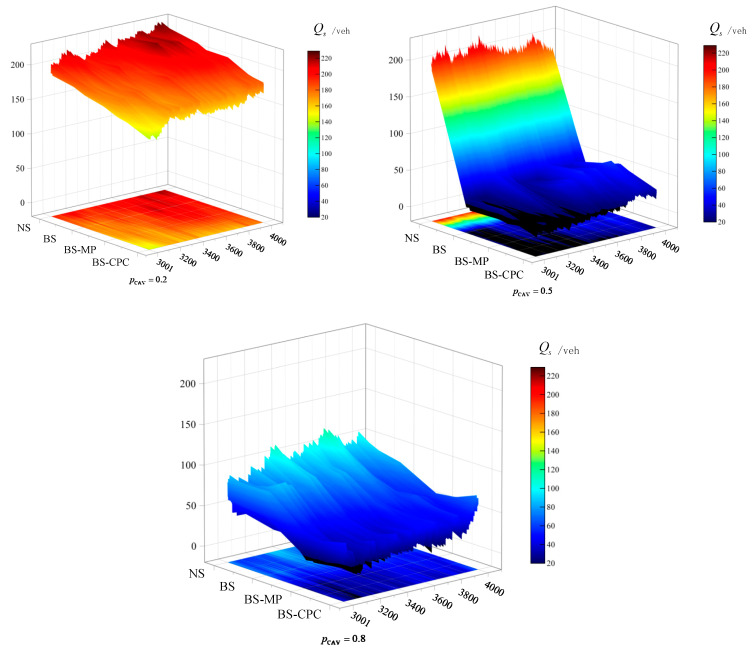
Heatmap of $Q_{s}$.

Then, we analyzed the vehicles experiencing congestion. According to [31], we defined a vehicle with a speed below 10 km/h as a severely congested vehicle and counted the number of such vehicles as $Q_{s}$. Figure 12 shows the distribution of $Q_{s}$ for each step in strategies NS, BS, BS-MP, and BS-CPC at different $p_{\mathrm{CAV}}$.

In Figure 12, regardless of $p_{\mathrm{CAV}}$, it can be seen that when under NS, the higher $p_{\mathrm{CAV}}$ indicates less congestion. When $p_{\mathrm{CAV}}=0.5$, the performance of road access was the best (either in BS, BS-MP, or BS-CPC) with the smallest value of $Q_{s}$. Moreover, the value of $Q_{s}$ decreased sequentially in NS, BS, BS-MP, and BS-CPC, which suggested that BS, BS-MP, and BS-CPC were beneficial in alleviating traffic congestion. At different $p_{\mathrm{CAV}}$, the value of $Q_{s}$ under BS-CPC is smallest. It is implied that CAV platoon cooperative lane changing is more conducive to road access compared to single-vehicle cooperation.

## 6. Conclusions

To ensure both bus priority and the full utilization of lane resources, a CAV control method of reusing bus lane is proposed. The CAV control of borrowing bus lanes considered the constraints of the moving gap between buses, while the returning control considered coordination with the nearest CAV platoon in the adjacent lane to cope with situations where CAVs cannot find the appropriate space. Four scenarios of when a CAV leaves the bus lane are analyzed, and the CAV platoon coordinative lane-changing algorithm is proposed to solve the problem where a CAV cannot find the safe and appropriate space to return. Subsequently, an open boundary cellular automaton model was established to simulate and compare the operating effects under the conditions of a single CAV (BS), moving gap constraints (BS-MP), and CAV platoon coordination (BS-CPC) for lane changing. The research results showed that CAV platoon coordinative lane changing could improve road traffic efficiently, and at different $p_{\mathrm{CAV}}$, the operating effect under CAV platoon coordinative lane changing control is optimal. When the average speed of the road is close to the average speed of the moving gap, the benefits of reusing bus lanes are not significant under BS-MP. When $p_{\mathrm{CAV}}=0.5$, the performance of road access is the best. In contrast, when the penetration rate is high, too many CAVs change to bus lanes, resulting in the inability to change out in a timely manner during congestion and affecting public transportation priority. When the penetration rate is low, fewer CAVs cannot fully utilize the bus lane, resulting in low sharing efficiency.

## Figures and Tables

**Table 1:** Parameter description table.

Notations	Description
N	Number of lanes. $l\in Ζ\left[1,N\right]$
$l_{c}(t)$	Clear distance of the bus at t; CAVs are forced to leave the bus lane within $l_{c}(t)$.
$d_{i,safe}(t)$	Safe distance of vehicle i at t, including two parts: reaction distance and braking distance.
$d_{i}(t)$	The distance between vehicle i and the preceding vehicle on the same lane at t.
$d_{i}^{\prime }(t)$	The distance between vehicle i and the preceding vehicle on the nearest lane at t.
$d_{i,back}^{\prime }(t)$	The distance between vehicle i and the following approaching vehicle on the nearest lane at t.
$a_{i}$	Acceleration, $a_{i}\in \left\{a_{\mathrm{BUS}},a_{\mathrm{HV}},a_{\mathrm{CAV}}\right\}$.
$b_{i}$	Deceleration, $b_{i}\in \left\{b_{\mathrm{BUS}},b_{\mathrm{HV}},b_{\mathrm{CAV}}\right\}$, taking a negative value.
τ	The reaction time of the driver, $\tau \in \left\{\tau_{\mathrm{BUS}},\tau_{\mathrm{HV}},\tau_{\mathrm{CAV}}\right\}$, $\tau_{\mathrm{CAV}}=0$.
$v_{i}(t)$	The speed of vehicle i at t.
$x_{i}(t)$	The location of vehicle i at t.
$x_{i}^{\prime }(t)$	The location of the adjacent lane corresponding to the location of vehicle i at t.
$v_{\max}$	Maximum speed, $v_{\max}\in \left\{v_{\max}^{\mathrm{BUS}},v_{\max}^{\mathrm{HV}},v_{\max}^{\mathrm{CAV}}\right\}$.
cells(x)	The cell state of $x_{i}(t)$.
Δt	Unit of time.
$p_{\mathrm{rand}}$	Randomization probability.
$\alpha_{i}$	Adaptive cruise control (ACC) following mode, CAV-HV or CAV-BUS; the value is 0 or 1.
$\beta_{i}$	Human-driving car following mode, HV-HV, BUS-BUS, or HV-CAV; the value is 0 or 1.
$\gamma_{i}$	Cooperative Adaptive Cruise Control (CACC) following mode, CAV-CAV; the value is 0 or 1.
$l_{\mathrm{cell}}$	Unit cell length.
$L_{\mathrm{HV}}$,$L_{\mathrm{BUS}}$,$L_{\mathrm{CAV}}$	The length of HV, BUS, and CAV, respectively.
$Q_{1-mp}$,$Q_{2-mp}$	The quantity of vehicles in the bus lane in the moving gap and the quantity of vehicles in the adjacent lane in moving gap.
${\overset{-}{v}}_{1-mp}$,${\overset{-}{v}}_{2-mp}$	The average speed in the bus lane in the moving gap and the average speed in the adjacent lane in the moving gap.

**Table 3:** Simulation parameter.

Name	Variable	Value	Unit
the length of road	L	2000 (2)	Cell (km)
number of lanes	N	2	-
time step	Δt	1	second (s)
acceleration	$a_{\mathrm{BUS}}$, $a_{\mathrm{HV}}$$a_{\mathrm{CAV}}$	3, 5, 5	m/s^2^
deceleration	$b_{\mathrm{BUS}}$, $b_{\mathrm{HV}}$, $b_{\mathrm{CAV}}$	−6, −8, −8	m/s^2^
The reaction time	$\tau_{\mathrm{BUS}}$, $\tau_{\mathrm{HV}}$, $\tau_{\mathrm{CAV}}$	0.4, 0.4, 0	second (s)
The length of vehicle	$L_{\mathrm{BUS}}$, $L_{\mathrm{HV}}$, $L_{\mathrm{CAV}}$,	10, 5, 5	cell (meters)
Maximum speed	$v_{\max}^{\mathrm{bus}}$, $v_{\max}^{\mathrm{HV}}$, $v_{\max}^{\mathrm{CAV}}$	14, 15, 15	cell/s (m/s)
Random slowing probability	$p_{\mathrm{rand}}$	0.2	
CAV penetration rate	$p_{\mathrm{CAV}}$		

**Table 4:** Detailed explanation of the four strategies.

Strategy	Explanation
BS	CAVs are allowed to use the bus lane. But the moving gap constraints are not considered. Additionally, CAV platoon collaborative lane changing is also not applied.
BS-MP	Based on BLIP-ST, the moving gap constraints are considered. CAV platoon collaborative lane changing is not applied, but single CAV collaborative lane changing is.
BS-CPC	Based on BLIP-ST, the moving gap constraints are considered, and CAV platoon collaborative lane changing is applied simultaneously.

## Data Availability

This study is a simulation study, and the key simulation parameters are listed in this paper. Regarding the raw data of the simulation results, this study is funded by the project, and the current project has not yet been completed; thus, the data cannot be disclosed for the time being.
